# Enhancing the accuracy, speed, and efficiency of kafirin‐PEO electrospun bio‐nanocomposite pH indicators with red beetroot extract using image processing

**DOI:** 10.1002/fsn3.3968

**Published:** 2024-01-16

**Authors:** Fatemeh Tavana, Abdollah Hematian Sourki, Mohammad‐Taghi Golmakani

**Affiliations:** ^1^ Department of Food Science and Technology, Faculty of Agriculture Jahrom University Jahrom Iran; ^2^ Department of Food Science and Technology, School of Agriculture Shiraz University Shiraz Iran

**Keywords:** betalain, biosensor, image processing, kafirin, red beetroot

## Abstract

Intelligent electrospun pH indicators were produced from bio‐nanocomposite kafirin‐polyethylene oxide (PEO) containing red beetroot extract. The aim was to evaluate the performance and stability of the electrospun pH indicators via image processing. Red beetroot extract was added to a mixture of kafirin and PEO at various concentrations. The mixtures were electrospun, and infrared Fourier transform spectroscopy confirmed the presence of kafirin, PEO, and red beetroot extract in the resulting pH indicator. The results showed that the pH indicators had high stability and reversibility at different temperatures, pHs, and environmental conditions. The results showed that the color of the indicators was significantly reversible after pH changes, with highly desirable reversibility observed at pH values of 1, 3, 4, 5, 7, 9, and 10. The findings proved that the red beetroot extract loaded bio‐nanocomposite pH indicator accompanied by evaluation of color characteristics through image processing technique, can serve as a time‐efficient, accurate tool for detecting and tracking pH changes caused by food spoilage.

## Graphical abstract

Intelligent pH indicators had high stability and reversibility at different temperatures, pHs, and environmental conditions. Image processing could increase the efficiency, accuracy, and speed of operation of the pH indicator. Using online image processing, the pH indicator can detect even the smallest changes in food spoilage that affect the pH.

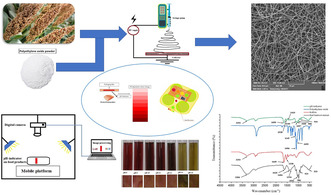

## 1 INTRODUCTION

In the past few decades, a substantial amount of research has been done on electrospinning to produce very thin fibers, ranging from 10 to several 100 nm in diameter. These nanofibers have had many applications in the food, pharmaceutical, and medical engineering industries (Bhushani & Anandharamakrishnan, 2014). Nanofibers obtained from electrospinning have many technological applications due to their unique features, such as high surface area‐to‐volume ratio, high length‐to‐diameter ratio, and the ability to create highly porous networks suitable for encapsulating bioactive compounds (Aman Mohammadi et al., 2020). In this regard, one of the applications of electrospun mats is to use them as bases for biological and chemical indicators and sensors. (Bhushani & Anandharamakrishnan, 2014; Ghorani & Tucker, 2015).

Microorganisms produce metabolites as they grow in food. The examination of these metabolites is directly related to the microbial growth rate. Counting the microbes and investigating their growth is time‐consuming and expensive, so the use of indicators that are sensitive to chemical reactions and metabolites resulting from the activity of microorganisms is a suitable method for quickly detecting their activities. A type of pH‐sensitive indicator is polymer films that change color when the pH of the environment changes. These indicators can be produced by two methods: electrospinning and casting (Greiner & Wendorff, 2007). One of the advantages of using electrospun nanofibers in the production of pH indicators is that, in addition to creating high porosity in the polymer network, these fibers also have a high surface area‐to‐volume ratio, which increases the responsiveness to changes in pH and reduces the response time to changes in environmental pH (Greiner & Wendorff, 2007).

Anthocyanins and betalains are among the best natural pigments in plants and are the most abundant natural pigments after chlorophyll. Anthocyanins and betalains have bioactive properties that make them antioxidants and anticancer agents (Váli et al., 2007). Anthocyanins and betalains are converted into different chemical forms outside the body of living organisms under the influence of pH, and, thus, they can change to different colors (Amnah, 2013; Azlim et al., 2022). These features offer incentives for researchers to extract anthocyanins and betalains while using them as healthy food colors in the food industry (Babaloo & Jamei, 2018). For example, due to the easy and simple, environment‐friendly, and economical extraction of betalain from beetroot, this pigment is a suitable option for the production of eco‐friendly mycological staining reagent (Sutradhar & Bhattacharya, 2021). Furthermore, they can be used in intelligent labels and smart packaging in formulations of indicators and biosensors, which are a relatively new field of research (Chausali et al., 2022; Otálora González et al., 2022; Remón et al., 2000). Red beetroot contains high amounts of flavonoid compounds and betalains [consisting betacyanins (red) and betaxanthins (yellow)]—the presence of which can increase antioxidant efficiency (Bahriye et al., 2023; Chandran et al., 2014). Compared to anthocyanins, betalains change color to a lesser extent, in response to variable pH. For this reason, betalains are less used in producing pH indicators. But since betalains are more stable against environmental conditions, such as temperature, highly acidic/alkaline pH values, and long storage time, they are potentially more applicable than anthocyanins (Montes‐Lora et al., 2016; Otalora et al., 2020). Thimmaraju et al. (2003) reported that red beetroot betalains have high stability in the range of pH 3–9, while Ekici et al. (2014) reported that the highest stability of anthocyanins was at pH 3, and with increasing pH from 3 to 7, the amount of anthocyanins decreased significantly. Therefore, Sui et al. (2014) believe that betalains (betacyanin and betaxanthin) are suitable alternative for anthocyanins in foods with acidic to neutral pHs. In addition, an essential difference between betalains and anthocyanins is that studies have shown that the betalains can regenerate after thermal treatment, for example, and this is not observed for anthocyanins (Cardoso‐Ugarte et al., 2014; Celli & Brooks, 2017; Khan & Giridhar, 2014).

Image processing techniques can assist in evaluating the changes in color characteristics of betalains against pH variations, thereby facilitating the use of these compounds in the structure of pH indicators for food application. Today, the electrospinning technique has been widely used in the production of natural and synthetic nanofibers due to its high speed, low cost, and high accuracy, so various researchers have used this technique to produce pH indicators from natural polymers (Aghaei et al., 2020; Nadi et al., 2023; Prietto et al., 2018), synthetic polymers (Agarwal et al., 2012; Moreira et al., 2018; Van der Schueren et al., 2010) and their composites (Forghani et al., 2022; Pakolpakçıl et al., 2018) containing different natural pigments and synthetic dyes. Although different natural pigments such as curcumin, quercetin, phycocyanin (Terra et al., 2021), alizarin, purpurin (Szadkowski et al., 2022), and *Citrus lanatus*, *Beta vulgaris*, and *Daucus carota* extracts (Ghatage et al., 2017) have been used as pH indicators, but the available literature contains no cases of research on the production of pH indicator kits containing red beetroot extract by the electrospinning method. Therefore, the purpose of this research was to produce bio‐nanocomposites containing red beetroot extract by the electrospinning method, and, thus, create pH indicators to track food spoilage. Also, it was aimed to evaluate the effectiveness of the image processing technique in testing the quality, performance, and stability of the pH indicator.

## 2 MATERIALS AND METHODS

### 2.1 Materials

Fresh red beetroots were purchased from local fruit shops. Grain sorghum (*Bicolor sorghum* L.) (Moench) was obtained from the Seed and Plant Improvement Institute (SPII), Karaj, Iran. PEO was purchased from Sigma‐Aldrich. Glacial acetic acid (100%), Tween 20, butanol, hydrochloric acid, sodium hydroxide, ethanol 96%, and sodium metabisulfite were obtained from Merck (Germany). Double distilled water was used to prepare the solutions.

### 2.2 Preparation of red beetroot extract

Red beetroot extract was prepared according to a method by Ben Haj Koubaier et al. (2014) with slight modifications. For this purpose, 100 g of red beets were crushed with a blender and mixed with 500 mL of distilled water. Then, the above mixture was stirred (100 rpm) for 24 h at room temperature. The obtained crude extract was centrifuged at 8000 rpm for 30 min, and the resulting supernatant was concentrated by a rotary evaporator. The concentrated beetroot extract was lyophilized by freeze dryer and maintained in polyethylene bags at −18°C (Ben Haj Koubaier et al., 2014).

### 2.3 Sorghum kafirin extraction

Kafirin protein was extracted from sorghum seeds, according to Emmambux and Taylor (2003). First, the sorghum seeds were cleaned by hand and then ground by a laboratory mill. Ethanol (70%) was used five times the weight of milled sorghum, along with sodium metabisulfite and sodium hydroxide which were added as solvents. This mixture was placed in a 70°C water bath and stirred for 1 h. Then, it was centrifuged at 1000 rpm for 5 min. After the centrifuge, the supernatant was separated and placed under the hood for 24 h. The sediments were separated and water was added as the equivalent weight of the sediments. The pH value reached 5 with 1 M hydrochloric acid. The liquid phase was removed through a Buchner funnel by a vacuum pump and filtered with Whatman filter paper No. 41. The concentrated material collected on a filter paper was dried in a hot‐air oven at 40°C and turned into a powder by a laboratory mill (Emmambux & Taylor, 2003).

### 2.4 Electrospinning process

The results of previous research showed that the optimal concentration of kafirin and PEO for the production of bio‐nanocomposite was 25% and 2%, respectively (Tavana et al., 2023). PEO solution 2% (w/v) and kafirin 25% (w/v) were prepared in glacial acetic acid and mixed at a ratio of 1:1. Then, the lyophilized red beetroot extract was added in amounts of 0.05, 0.15, 0.3, 0.4, 0.5, 0.75, and 1.5 g to 10 mL of the above mixture. They were stirred on magnetic stirring 90 min at room temperature to obtain homogeneous solutions. The final solutions were electrospun at a voltage of 20 kV, a distance of 150 mm, and a flow rate of 0.6 mL/h.

### 2.5 Scanning electron microscope (SEM) analysis of electrospun bio‐nanocomposite

After producing the nanofibers by electrospinning, their morphology and nanostructure were imaged and analyzed by a scanning electron microscope (SEM) (TESCAN‐VEGA3, TESCAN‐Cesco Republica). The nanofibers were dried in a vacuum for 24 h, were separated from the aluminum foil, and covered with gold coating before being scanned (Q150R‐ES, Quorum Technologies). All observations were made through magnifications that ranged from 1000× to 2000×, and SEM images assisted in selecting optimal fibers from the electrospun samples.

### 2.6 Fourier transform infrared (FT‐IR) analysis

Almost all compounds with covalent bonds absorb different frequencies of electromagnetic radiation in the infrared region. The FT‐IR test was used to evaluate the interaction between kafirin biopolymers, PEO, and red beetroot extract during the electrospinning process. Also, it was tested to prove the presence of all three compounds in the resultant bio‐nanocomposite. The kafirin, PEO, and red beetroot powders were ground with potassium bromide (KBr) and then pressed into pellets before measurement. The FT‐IR operated using a Bruker Tensor II FT‐IR device at the frequency range of 400–4000 cm^−1^ at a resolution of 1.43 cm^−1^(Ali et al., 2014). The interference of H_2_O and CO_2_ in the air was subtracted during analysis.

### 2.7 Image processing and pH indicator colorimetry analysis

A photographic box was used for photographing the pH indicators. A digital photography camera (Canon Powershot A495) was installed on a horizontal stand connected to a wall. It was completely perpendicular to the place where the box containing the sample was situated. The camera could zoom 3× to 6×, with a digital zoom of 4×, a sensor resolution range of 10–12 megapixels, and a screen size of 2.5 inches. Samples of the pH indicator, based on kafirin‐PEO bio‐nanocomposite, were placed at a distance of 18 cm from the camera lens. The camera was set to zoom 2×. The images were analyzed by ImageJ software. RGB and L*, a*, and b* factors were calculated and a graph of data variation was illustrated by Microsoft Excel 2016. Also, the total color differences were measured and compared with each other according to the following equation:

(1) $$∆E=\sqrt{{\left(L_{2}-L_{1}\right)}^{2}+{\left(a_{2}-a_{1}\right)}^{2}+{\left(b_{2}-b_{1}\right)}^{2}}$$

### 2.8 Thermal stability of the pH indicator at different pH and temperatures

The thermal stability of the pH indicator was evaluated at pH values of 2, 5, and 10, at −18°C and 100°C, according to Devarayan and Kim (2015). Six mats of kafirin‐PEO bio‐nanocomposite containing red beetroot extract were used. Using hydrochloric acid and sodium hydroxide, the solutions were adjusted at appropriate pH values (2, 5, and 10) and were poured on the pH indicator mats using a dropper. Then, the mats were dried at room temperature. Three of the mats were kept at −18°C for 24 h, and the other three were kept in a hot‐air oven (100°C) for 24 h. Photographs of the pH indicator mats were taken before, and after pH application and storage at different temperatures. To check the thermal stability of pH indicators, their color parameters, including the intensity of red, green, and blue colors in the RGB color space, as well as the L*, a*, and b* parameters in the LAB color space, were determined and compared.

### 2.9 Stability of the pH indicator in aqueous solutions

A pH indicator mat was placed in distilled water for 24 h and then dried at room temperature (Devarayan & Kim, 2015). The stability of the pH indicator in aqueous solutions was determined and compared with RGB and LAB color spaces, before, and after the exposure to distilled water.

### 2.10 Reversibility of the pH indicator

The reversibility and reusability of the pH indicator were evaluated at pH values of 1–14. First, solutions of 1–14 pH values were made by hydrochloric acid and sodium hydroxide. These solutions were poured onto the pH indicator using a dropper. Then, all pH indicators were dried at room temperature. To check the reversibility of the pH indicators, the pH 2 solution was added to all indicators by a dropper and then allowed to dry at room temperature. Again, the pH values of 1–14 were applied to the indicators for a second time and allowed to dry at room temperature. Tests were done to determine the reversibility and reusability of the pH indicators by imaging them before and after applying the pH values of 1–14, after applying pH 2, and finally after applying the pH values of 1–14 again for the second time (Devarayan & Kim, 2015; Maftoonazad & Ramaswamy, 2019).

### 2.11 Statistical analysis

Statistical analyses were performed to evaluate the color space values. One‐way analysis of variance (ANOVA) was applied to the data using the statistical software, Minitab.20.3.0.0 (Minitab Inc, UK). A *p* ≤ .05 was considered significant.

## 3 RESULTS AND DISCUSSION

### 3.1 Morphological studies of electrospun bio‐nanocomposite

**FIGURE 1:**
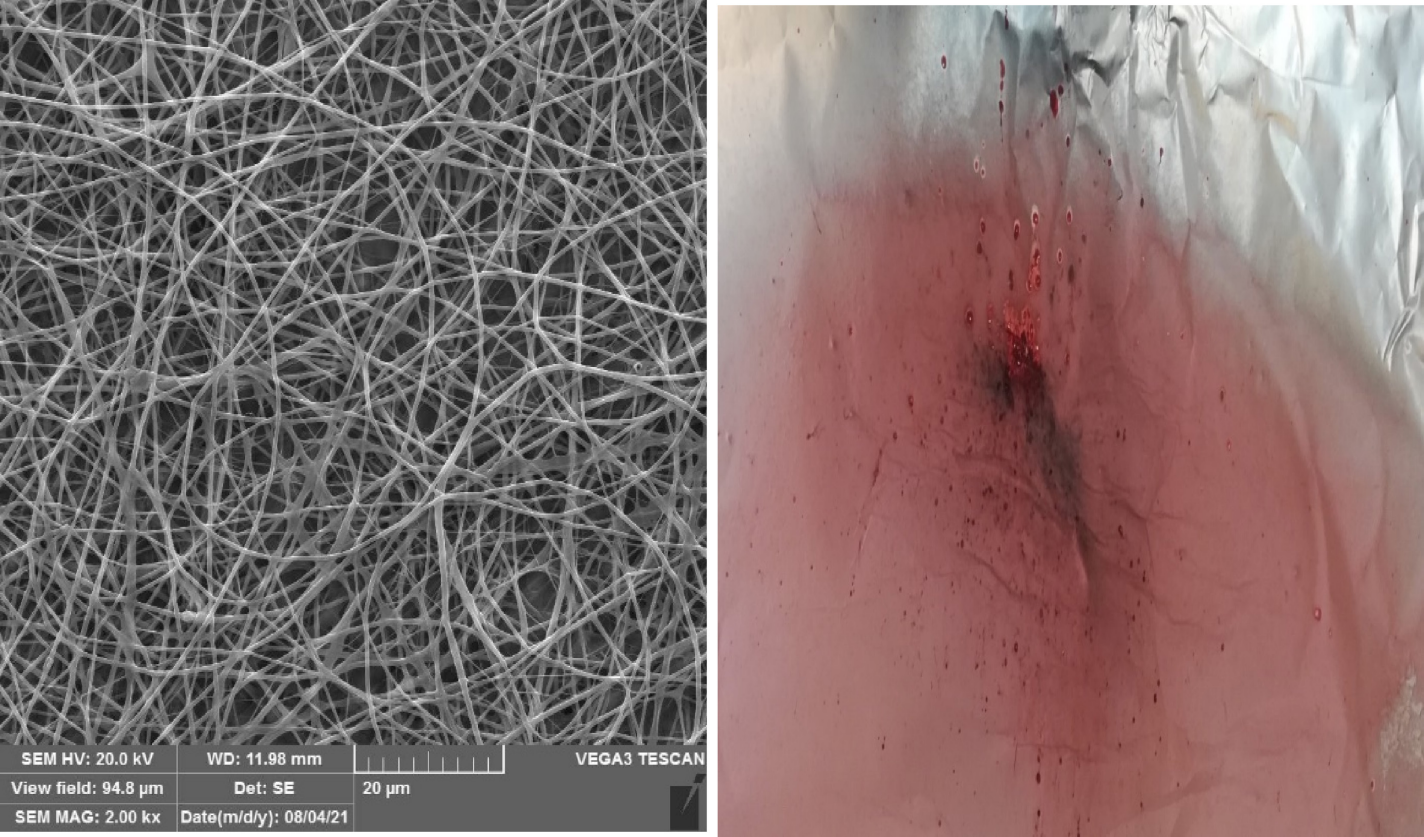
(a) SEM image of electrospun nanofibers of kafirin/PEO at optimal concentration; (b) kafirin/PEO nanocomposite as pH indicator containing red beetroot extract (15%).

Previous results of relevant research showed that the best concentration of kafirin and PEO for the production of bio‐nanocomposite pH indicators was 25 and 2% (w/v), respectively, in glacial acetic acid (Tavana et al., 2023). Figure 1a demonstrates the structure of kafirin‐PEO bio‐nanocomposite. The evaluation of SEM images showed that nanofibers with appropriate morphological characteristics and without beads were formed in those concentrations. Accordingly, the resultant nanofibers had average diameters of 220.51 ± 26.6 nm. The uniformity of the nanofiber surface and low mean diameter increased the nanofibers' surface area‐to‐volume ratio. It caused a uniform distribution of the colored extract onto the entire surface of the pH indicator. Visual observations revealed that the intensity of the red color significantly increased in the electrospun mats when higher concentrations of red beetroot extract were used (*p* < .05). Accordingly, a concentration of 15% red beetroot extract was selected as an optimal concentration as it caused a greater color intensity than the other concentrations (Figure 1b).

### 3.2 Fourier transform infrared spectroscopy (FT‐IR)

**FIGURE 2:**
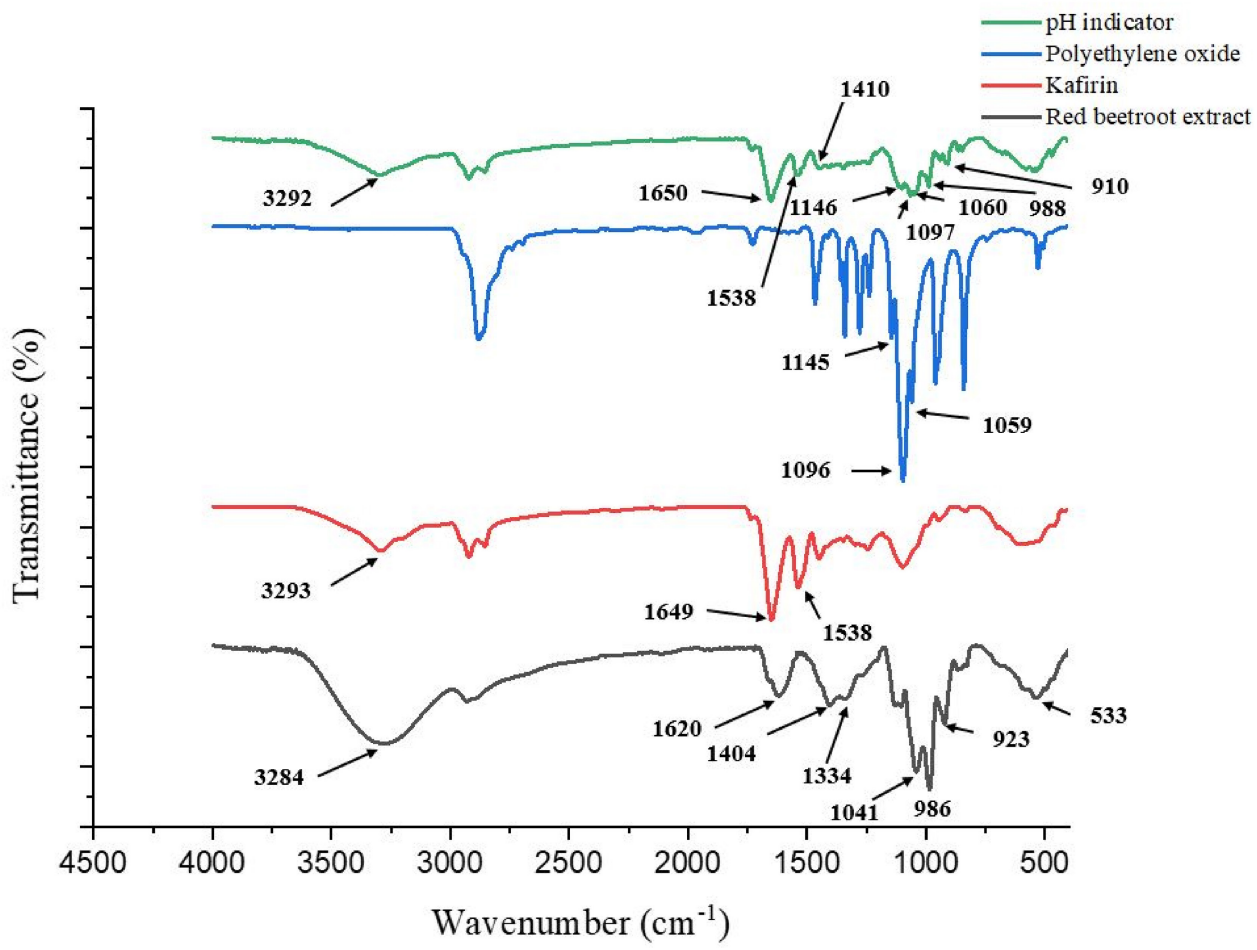
Fourier transform infrared spectrum of pH indicator and its components.

The results of Fourier transform infrared spectroscopic analysis of red beetroot extract showed that the pigments of this extract have several significant absorption peaks in wavelengths of 3284, 1620, 1404, 1334, 1041, 986, 923, and 533 cm^−1^, which indicated O‐H stretching bond, C=N stretching vibration, C‐H deformation, C‐COOH stretching bond, C‐O stretching bond, C‐H deformation, C‐H deformation, and C‐O‐Si stretching bond, respectively (Figure 2). Molina et al. (2014) reported that the FT‐IR spectrum of *Beta vulgaris* extract has absorption peaks in wavelengths of 1624, 1402, 1320, 990, 915, and 530, respectively, indicating C=N stretching vibration, C‐H deformation, C‐COOH stretching bond, C‐H deformation, and C‐O‐Si stretching bond. Venugopal et al. (2017) reported that the FT‐IR spectrum of *Beta vulgaris* extract have the absorption peaks in wavelengths of 3237–3565, which indicates the O‐H stretching bond. The results of the FT‐IR spectrum analysis of the pH indicator, containing red beetroot extract, showed that it has prominent peaks in wavelengths of 3292, 1410, 988, 910, and 534.21 cm^−1^ (Figure 2). The FT‐IR spectrum of the pH indicator showed absorption peaks that closely resembled those of pure red beetroot extract FT‐IR spectrum. This similarity among the peaks of wavelengths indicates the presence of red beetroot extract in the pH indicator nanocomposite. Also, it meant that the beetroot extract did not react chemically with the kafirin and PEO biopolymers.

The infrared Fourier transform spectrum of the pH indicator had three vibrations in the wavelengths 3292, 1650, and 1538 cm^−1^, which correspond to the stretching vibration of the hydrogen bond of the N‐H group, the stretching vibrations of C=O, and the bending vibration of the amide bond in the N‐H group (Figure 2). These bonds prove the existence of kafirin in electrospun nanofibers in the pH indicator structure. In similar results, Xiao et al. (2016) attributed the presence of three absorption peaks at wavelengths 3290, 1653, and 1538 cm^−1^ in the FT‐IR spectrum of kafirin to the stretching vibration of the hydrogen bond in the N‐H group, the stretching vibrations of C=O, and the bending vibration of the amide bond in the N‐H group.

The FT‐IR spectrum analysis of the pH indicator showed that three absorption peaks at wavelengths of 1146, 1097, and 1060 cm^−1^ were clearly observed, which are probably related to the C‐O‐C stretching vibration in the PEO structure (Figure 2). Aluigi et al. (2007) reported that the Fourier transform infrared spectrum of pure PEO showed three absorption peaks at 1145, 1094, and 1060 cm^−1^ wavelengths, which are related to the C‐O‐C stretching vibration. The C‐O‐C stretching vibration showed absorptions in the range of 1050–1150 cm^−1^, which are generally observed as three absorption peaks in this region (Aluigi et al., 2007). The presence of absorption peaks in the FT‐IR spectrum of kafirin‐PEO bio‐nanocomposite, containing red beetroot extract, proved the presence of kafirin and PEO in this nanocomposite. Also, the lack of significance variation in the location of these peaks in the FT‐IR spectrum of the pH indicator indicated the absence of chemical interactions between kafirin, PEO, and red beetroot pigments. Thus, it can be concluded that these components were physically present in a composite structure and are safely compatible with each other.

### 3.3 Color characteristics analysis of pH indicator

**FIGURE 3:**
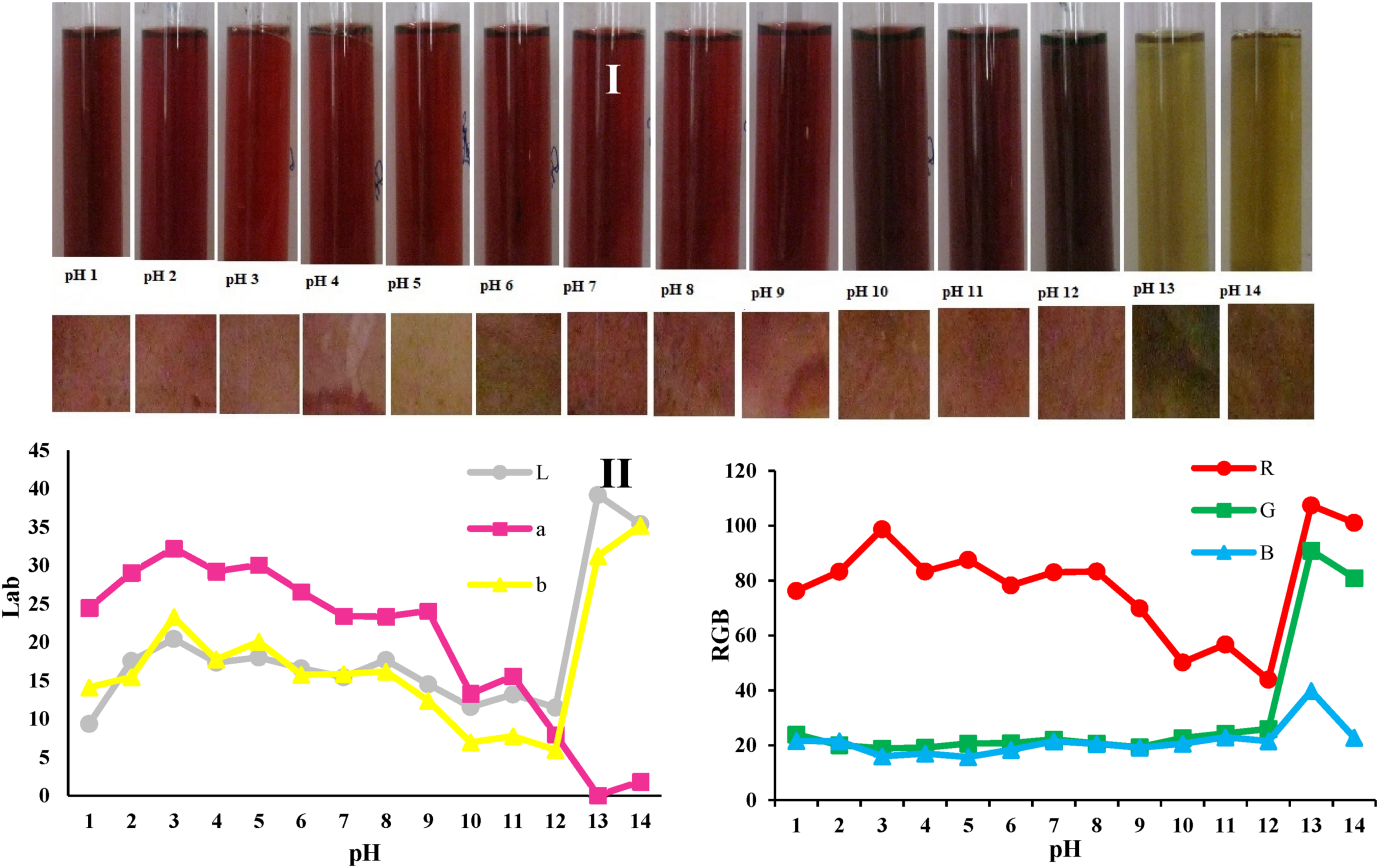
(I) Color patterns of red beetroot extract solution and pH indicator nanocomposite mats at pH 1–14. (II) The Lab and RGB values of pH indicator mats after pH treatment.

After adding solutions with pH 1–14 to the pH indicator, color changes were observed after a few seconds in the respective pH values (Figure 3). By applying different pH values, red color was observed of the indicators impregnated with solutions of pH 1–12, with different intensities, whereas the samples impregnated with solutions of pH 13 and 14 showed a greenish‐yellow color (Figure 3). Maftoonazad and Ramaswamy (2019) used polyvinyl alcohol polymer and added red cabbage extract to it, thereby producing nanofibers by electrospinning and using them as pH indicators. In acidic environments, with a pH <3, the pH indicators showed a red color, and, by increasing the pH, the color became blue and yellow. The color of betalain is not the same as that of anthocyanins in relation to pH, and, thus, to compare the effect of pH on the performance of the pH indicator, their color characteristics were examined and compared by image processing technique. The results showed that with the increase in pH from 1 to 3, the color indexes of L*, a*, and b* increased significantly (*p* < .05), but then with the increase in the pH to 12, these indexes decreased significantly and led to a decrease in lightness and redness, but an increase in yellowness (Figure 3). With the rise in pH from 12 to 14, the values of L* and b* increased significantly (*p* < .05), but the value of the a* decreased. At pH values over 12, the lightness and yellowness increased significantly (*p* < .05), but the redness decreased (Figure 3). The amount of changes in the R index (redness) was evaluated at pH 1–14, and the results demonstrated that the redness color (R index) was more intense in acidic pH values. The intensity of redness decreased in response to the pH rise to 12. At pH 13, this amount increased again and revealed the most intense redness at pH 13 (Figure 3). With the increase in pH, the intensity of the G index (greenness) increased significantly (*p* < .05). The lowest and highest intensities of G were observed at pH values of 3 and 13, respectively. With the increase in pH, the intensity of blue color (B index) increased insignificantly (*p* > .05) (Figure 3). The significant changes in color characteristics of the RGB and the L*, a*, and b*, changes in the pH values of food can be easily detected by monitoring the results of image processing in the electrospun pH indicators containing red beetroot extract.

Using L*, a*, and b* values, the total color difference (∆*E*) for nanocomposite mats at each pH was calculated with respect to other pHs. The results showed that the ∆*E* values between different pHs ranged from a minimum of 2.93 (between pH 6 and 8) to a maximum of 24.36 (between pH 5 and 7) (Table 1). Considering that the ∆*E* values for kafirin‐PEO nanocomposite containing red beetroot extract were not very high. In most cases, the color difference was seen with the naked eye, it seems that to reduce the vision error and increase the accuracy and speed of color processing, it is better to use image processing techniques, especially online image processing to distinguish the colors obtained from different pHs. Devarayan and Kim (2015) reported ∆*E* values for cellulose nanofibers containing red cabbage extract at different pHs between 8 and 112. They believe that the high ∆*E* values make it easy to detect the color difference at different pH levels with the naked eye. However, they also used the image processing technique and comparison of *L*, a*, and b* indices to accurately distinguish between the samples of cellulose nanofibers containing red cabbage extract.

**TABLE 1:** Δ*E* values of kafirin‐PEO electrospun bio‐nanocomposite mats at each pH with respect to other pHs.

pH	1	2	3	4	5	6	7	8	9	10	11	12	13	14
2	5.01													
3	9.91	May‐30												
4	6.88	3.95	4.36											
5	18.21	13.72	8.51	12.29										
6	15.72	15	13.75	14.09	15.03									
7	9.6	13.79	17.45	14.56	24.36	14.9								
8	14.45	12.93	11.11	12.02	12.26	2.93	15.46							
9	14.09	9.17	4.76	9.07	5.82	15.77	21.58	12.86						
10	6.44	4.45	7.04	7.21	13.99	12.55	12.6	10.64	9.84					
11	7.92	4.35	4.81	6.22	11.49	12.15	14.51	9.81	7.41	2.53				
12	11.47	7.21	2.5	4.91	7.63	14.26	18.76	11.65	5.37	9.35	7.17			
13	17.93	18.34	17.76	17.18	19.46	4.89	14.54	7.7	20.32	16.13	16.16	18.9		
14	15.1	15.25	15.25	15.28	17.78	4.17	12.79	17.41	11.98	12.37	16.33	5.89		

### 3.4 Thermal stability of the pH indicator

#### 3.4.1 Effect of high temperature on color characteristics

**FIGURE 4:**
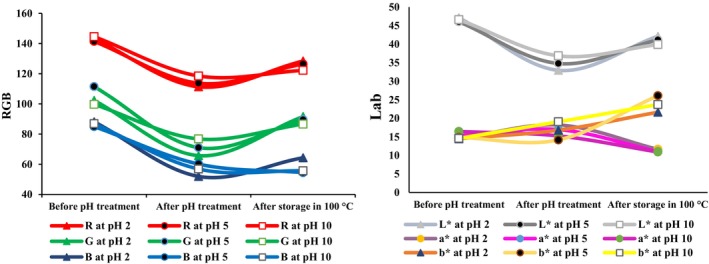
Changes in RGB and Lab values of pH indicator mats treated by different pH and storage at 100°C for 24 h.

The changes in R, G, and B color values after storage at 100°C for 24 h are presented in Figure 4. The results showed that by adding solutions with pH values of 2, 5, and 10 to the pH indicator, and after the mats were dried, the intensity of the red color (R index) decreased in all the samples. While storing the pH indicators at 100°C for 24 h, the intensity of the red color (R index) increased in three of the mats at pH 2, 5, and 10 (Figure 4). At high temperatures, the Maillard reaction and caramelization were more likely, resulting in the production of brown pigments. The R index increased during storage at 100°C, probably because of the formation of brown pigments caused by these nonenzymatic browning reactions (Patras et al., 2010).

Similar to the changes in the R index, after adding different pH solutions to the indicators, the intensity of the green color (G index) decreased. While storing the indicators at 100°C, the G parameter significantly increased in all three mats at pH 2, 5, and 10 (*p* < .05). Also, the intensity of the blue color (index B) significantly increased from 51.91 to 64.29 at pH 2 and decreased from 60.12 to 54.44 and from 56.82 to 55.64 at pH 5 and 10, respectively (Figure 4). The changes in colors resulted from changes in the structure of anthocyanins and betalains in the red beetroot extract, which are influenced by several factors, mainly variations in pH and temperature (Patras et al., 2010).

Color analysis can be done either manually or automatically. Color analysis by the naked eye can be subjective and usually varies significantly from person to person. To make a color analysis, color standards are typically used as a reference. In this research, the LAB color system, and the L*, a*, and b* indexes were used to evaluate color changes of the pH indicator. The indices of this system, especially the L* index, are closely related to the human perception of lightness. L* is the lightness index and varies between 0 and 100. Parameter a* varies from green to red, and b* from blue to yellow. Both color components range from −120 to 120 (Mendoza et al., 2006).

The stability of the pH indicator at 100°C after 24 h was measured by examining the changes in L*, a*, and b* values (Figure 4). The results showed that after using solutions of pH 2, 5, and 10 on the pH indicators, the lightness of the color decreased significantly. By placing them at 100°C, the lightness (L* index) of the pH indicators at pH 2, 5, and 10 significantly increased from 32.95 to 42.12, from 34.78 to 41.16, and from 36.85 to 39.89, respectively (*p* < .05). These changes indicate an apparent increase in the lightness of the pH indicator (Figure 4). At high temperatures, pigments oxidize faster. Many anthocyanins and betalain pigments are destroyed at high temperatures. In particular, the destruction of betalain compounds in red beetroot extract at high temperatures causes an increase in the lightness of the pH indicator (Patras et al., 2010).

By exposing the pH indicator to 100°C, the a* index significantly decreased from 18.21 to 11.63, from 16.97 to 11.02, and from 15.26 to 10.92 in response to the solutions of pH 2, 5, and 10, respectively (*p* < .05). The b* index in the pH indicators at 100°C significantly increased from 16.79 to 21.64, from 14.16 to 26.10, and from 19.05 to 23.72 in response to the solutions of pH 2, 5, and 10, respectively (*p* < .05). These changes are probably caused by the effect of pH, temperature, light, oxygen, enzymatic decomposition and interactions with other substances such as sugars, metal ions, and other pigments (Patras et al., 2010).

#### 3.4.2 Effect of low temperature on color characteristics

**FIGURE 5:**
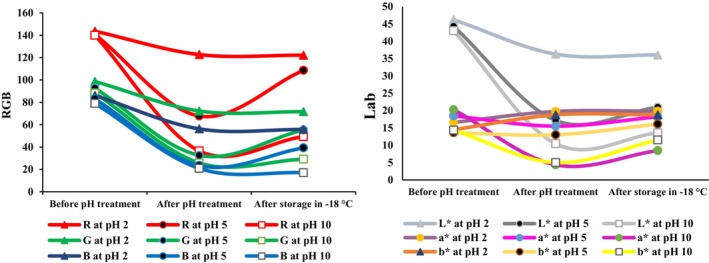
Changes in RGB and Lab values of pH indicator mats treated by different pH and storage at −18°C for 24 h.

Changes in R, G, and B color values after storage at −18°C for 24 h are shown in Figure 5. After using solutions of pH 2, 5, and 10 at −18°C, the redness (R index) at pH 2 decreased from 122.76 to 122.22, which was not significant. At pH 5 and 10, the R index significantly increased from 67.82 to 108.7 and from 36.56 to 49.41 (Figure 5; *p* < .05). The green color (G index) insignificantly decreased from 72.29 to 71.68 at pH 2. However, it increased from 70.98 to 89.49, and from 76.77 to 86.58 at pH 5 and 10, respectively (Figure 5). The blue color (B index) significantly increased from 51.92 to 64.29 at pH 2, but decreased from 60.12 to 54.44, and from 56.82 to 55.64 at pH 5 and 10, respectively (Figure 5).

The stability of the pH indicator at −18°C after 24 h was monitored (Figure 5). After using pH values of 2, 5, and 10 and storing them at −18°C, the lightness (L*) at pH 2 decreased insignificantly and changed from 36.29 to 36.06, but at pH 5 and 10, the lightness significantly increased from 17.16 to 20.97, and from 10.45 to 13.56, respectively (Figure 5) (*p* < .05). According to Figure 5, the slightest change in lightness (L*) was observed at pH 2, which indicates the greater stability of anthocyanin and betalain compounds in acidic pH solutions. The changes in L* increased in association with the increase in pH, which means a decrease in the stability of anthocyanin and betalain compounds in the red beetroot extract against alkaline pH solutions (Wahyuningsih et al., 2017). The a* index increased from 19.76 to 19.83, from 15.50 to 18.21, and from 4.35 to 8.47, at pH values of 2, 5, and 10, respectively. However, the change was not significant in pH 2 (Figure 5). The b* index slightly decreased from 18.77 to 18.76 at pH 2, which was not significant. At pH 5 and 10, these values increased from 13.01 to 16.11 and 4.99 to 11.53, respectively (Figure 5).

The stability of the pH indicator at −18°C and 100°C was calculated by measuring the total color difference (∆*E*) (Table 2). The stability of the pH indicator at −18°C was higher than at 100°C because the rate of anthocyanin decomposition increased in response to higher temperatures (Scalzo et al., 2008). The mutual effect of increasing temperature and alkaline pH probably causes more instability and degradation of the pigments, which causes more color change in the pH indicator. Meanwhile, the increase in temperature and the pH value may increase the rate of browning reactions and the formation of dark pigments in the structure of the pH indicator, thereby increasing the amount of the total color difference (∆*E*). Devarayan and Kim (2015) used cellulose polymer and added red cabbage extract to produce electrospun nanofibers that acted as pH indicators. To evaluate the stability of this pH indicator, they placed the made mats at different temperatures (100°C, −50°C) and reported no significant change in color at −50°C, meaning that the mats maintained their original state. At 100°C, however, the color patterns of the mats differed from their original form. In other words, the increase in temperature accelerated pigment decomposition.

**TABLE 2:** Total color difference (∆*E*) of the electrospun bio‐nanocomposite pH indicator at different conditions.

pH	∆*E*			
	100°C	−18°C	30 days storage at room temperature	24 h immersion in distilled water
2	12.28	0.24	—	—
5	14.78	5.60	—	—
7	—	—	7.77	13.92
10	7.05	8.32	—	—

#### 3.4.3 Effect of room temperature on color characteristics

**FIGURE 6:**
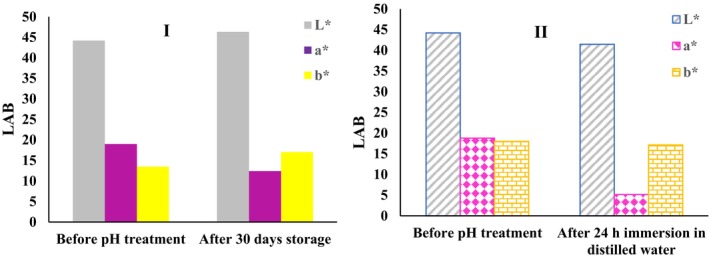
The stability of pH indicator nanocomposite: (I) at room temperature, (II) in aqueous medium.

The kafirin‐PEO bio‐nanocomposite, containing red beetroot extract, was stored for 1 month at room temperature. The evaluation of stability is shown in Figure 6. The intensity of the L* index and the b* index increased, whereas the a* index decreased from 18.99 to 12.40 (Figure 6). These changes were not significant considering 1 month, meaning that the pH indicator had good stability at room temperature.

The results of the total color difference (∆*E*) showed that the pH indicator containing red beetroot extract had relatively good stability after storage for 1 month at room temperature. Changes in the values of the total color difference (∆*E*) in 1 month of storage were very small (Table 2). Devarayan and Kim (2015) stated that by placing the pH indicator mats at room temperature for more than 1 month, no significant changes occurred in the ∆*E* value, indicating good color stability during storage.

### 3.5 Stability of pH indicator in an aqueous medium

Kafirin‐PEO bio‐nanocomposite, containing red beetroot extract, was immersed in distilled water for 24 h. The results showed that the lightness value (L* index) decreased from 44.22 to 41.48. Also, the *a index and b* index decreased (Figure 6). The changes in a* and b* values, indicating green‐red and blue‐yellow colors, were significant compared to the changes in L*. Red beetroot betalain pigments are mostly soluble in water and easily dissolve in an aqueous environment. However, PEO is soluble in water and cannot prevent water from entering the bio‐nanocomposite structure and dissolving pigments. Therefore, by placing the pH indicator in an aqueous environment, large amounts of betalain compounds are dissolved and can enter the aqueous phase, thereby causing a decrease in the a* and b* values.

The pH indicator containing red beetroot extract was not stable after 24 h in distilled water, so the changes in ∆*E* showed that part of the pigments were removed from the structure of the pH indicator due to dissolution in the aqueous environment (Table 2). Devarayan and Kim (2015) reported that the pH indicator containing red cabbage extract was immersed in distilled water for 24 h, where approximately 40% of the pigments were dissolved in water within 24 h. The total color difference (∆*E*) was relatively high, which indicates the low stability of this type of indicator in aqueous environments.

### 3.6 Reversibility and reusability of pH indicator

The reversibility of the pH indicator was a measure of the total color difference values (∆*E*) before and after applying the different treatments. The reversibility of the pH indicator in response to changes in pH exhibited significant differences in ∆*E* values across various pH levels. (Table 3). Thus, different pH values in the environment can be recognized by calculating the color parameter changes of the pH indicator. If the value of ∆*E* is greater than 5, the colors are distinguishable, whereas if the value of ∆*E* is greater than 12, the color becomes attributed to a different space (Agarwal et al., 2012). To evaluate the reversibility of pH indicators, the color difference (∆*E*) was calculated before changing the pH of pH indicators to 2 and when returning to their initial pH. The results showed that the pH indicator was mostly reversible when changing from a specific pH to pH 2, regardless of the initial pH (Table 3). This means that the pH indicator of kafirin‐PEO containing red beetroot extract is highly reversible against changes in pH.

**TABLE 3:** Evaluating the reversibility of pH‐sensitive bio‐nanocomposites at different pH values.

pH	1	2	3	4	5	6	7	8	9	10	11	12	13	14
∆*E*	4.2	9.04	3.76	5.23	2.44	8.62	2.39	8.81	5.16	3.74	5.24	7.49	12.04	8.43

## 4 CONCLUSION

The purpose of this research was, firstly, to use sorghum kafirin nanofibers with PEO to produce bio‐nanocomposites containing red beetroot extract. Thess bio‐nanocomposites can be used as a pH indicator to monitor food spoilage. The second aim of this research was to determine the feasibility of using image processing to render evaluations time‐efficient for measuring the performance and stability of the formulated pH indicator. In this research, the pH indicator changed color in response to all pH values, and, thus, can detect food spoilage by revealing changes in pH. This indicator has good reversibility and can be used several times without a decrease in efficiency (reversibility and reusability). Also, the stability of the pH indicator showed relatively good resistance to extreme temperatures and environmental conditions. Nonetheless, it is susceptible to aqueous environments because of the PEO water‐soluble polymer. In general, this pH indicator can be used in association with image processing to identify pH changes in foods that need to be checked for spoilage.

## AUTHOR CONTRIBUTIONS

**Fatemeh Tavana:** Data curation (equal); formal analysis (equal); methodology (equal); writing – original draft (equal). **Abdollah Hematian Sourki:** Conceptualization (lead); investigation (lead); methodology (lead); project administration (lead); resources (equal); supervision (lead); validation (lead); writing – original draft (equal); writing – review and editing (lead). **Mohammad‐Taghi Golmakani:** Funding acquisition (supporting); investigation (supporting); methodology (supporting); project administration (supporting); resources (lead); supervision (supporting).

## FUNDING INFORMATION

The author(s) received no financial support for the research, authorship, and/or publication of this article.

## CONFLICT OF INTEREST STATEMENT

The authors declare that they have no known competing financial interests or personal relationships that could have appeared to influence the work reported in this article.

## ETHICS STATEMENT

This study does not involve any human or animal testing.

## Data Availability

The data that support the findings of this study are available from the corresponding author upon reasonable request.
